# Practical tips to using formalin-fixed paraffin-embedded tissue archives for molecular diagnostics in a South African setting

**DOI:** 10.4102/ajlm.v11i1.1587

**Published:** 2022-06-23

**Authors:** Barbara S. van Deventer, Lorraine du Toit-Prinsloo, Chantal van Niekerk

**Affiliations:** 1 Department of Forensic Medicine, Faculty of Health Sciences, University of Pretoria, Pretoria, South Africa; 2 Department of Chemical Pathology, Faculty of Health Sciences, University of Pretoria, Pretoria, South Africa

**Keywords:** Autopsy, deoxyribonucleic acid, formalin-fixed paraffin-embedded tissue, formalin-fixed paraffin-embedded tissue archive, high-resolution melt analysis, molecular diagnostics, polymerase chain reaction, post-mortem genetic testing, sequencing

## Abstract

**Background:**

Formalin-fixed paraffin-embedded (FFPE) tissue archives in hospitals, biobanks, and others offer a vast collection of extensive, readily available specimens for molecular testing. Unfortunately, the use of tissue samples for molecular diagnostic applications is challenging; thus, the forensic pathology FFPE tissue archives in Africa have been a largely unexploited genetic resource, with the usability of DNA obtainable from these samples being unknown.

**Intervention:**

The study, conducted from January 2015 to August 2016, determined the usefulness of FFPE tissue as a reliable source of genetic material for successful post-mortem molecular applications and diagnostics. Formalin-fixed paraffin-embedded tissue samples were collected and archived from autopsies conducted over 13 years in the forensic medicine department of the University of Pretoria (Pretoria, South Africa). Deoxyribonucleic acid from FFPE tissue samples and control blood samples was amplified by high-resolution melt real-time polymerase chain reaction before sequencing. The procurement parameters and fixation times were compared with the quantity and quality of the extracted DNA and the efficiency of its subsequent molecular applications.

**Lessons learnt:**

This study has shown that FFPE samples are still usable in molecular forensics, despite inadequate sample preparation, and offer immense value to forensic molecular diagnostics.

**Recommendations:**

FFPE samples fixed in formalin for more than 24 h should still be used in molecular diagnostics or research, as long as the primer design targets amplicons not exceeding 300 base pairs.

## Background

Advances in molecular biology have enabled the detection of preventable and treatable diseases at a genetic level and it is a field of study receiving growing attention over the years.^1,2,3^ Globally, formalin-fixed paraffin-embedded (FFPE) tissue samples are the largest available and most used source of biological material for molecular applications – diagnostic purposes or research.^3,4,5^ Formalin-fixed paraffin-embedded tissue archives in hospitals, private pathology institutions, biobanks, and tertiary academic pathology departments offer a vast collection of extensive, readily available specimens for molecular testing.^6,7,8^ The critical information these specimens contain and their undeniable usefulness to necessary health-related investigations are considered by many researchers to be invaluable for molecular diagnostics.^4,8,9^

Unfortunately, the low quality and efficiency of DNA extracted from FFPE samples in molecular diagnostic applications limits the use of archived FFPE tissue samples.^10,11,12^ Potential factors influencing the limited molecular utility include different parameters used in the procurement, fixation and processing of these samples.^1,6,11^ Particularly, formalin fixation causes DNA fragmentation, formaldehyde exposure causes DNA-protein cross-linking, and paraffin in DNA samples inhibits polymerase chain reaction (PCR) amplifications. These factors impair the DNA quality and efficiency for molecular applications, such as PCR and sequencing.^9,11,12,13^ Although blood collected in ethylenediaminetetraacetic acid or fresh frozen tissue samples are the best source of DNA and the preferred material used for genetic testing, numerous studies have reported on the successful use of FFPE tissue samples.^2,14,15^ The successful use of FFPE tissue samples is particularly encouraging because African forensic pathology still faces the dire reality of inadequate funding and resource allocation required to maintain freezer preservation systems for routine collection and storage of blood or tissue samples.^15,16,17^

Forensic molecular pathology is an emerging field with significant clinical impact; its techniques are used to diagnose preventable and inherited causes of death.^18,19,20^ The role of the forensic pathologist in the medico-legal investigation of death includes determining the cause and manner of death. Despite the implementation of forensic molecular investigations and research in many high-income countries, there is still a significant lack of it in low- and middle-income countries, mainly due to financial and resource constraints.^9,15,21^

Several South African universities’ forensic medicine departments conduct valuable research, mainly focusing on possible causes of sudden deaths; however, there is still a remarkable paucity of publications on molecular forensic studies and their applications in Africa.^22,23,24^ Social and medico-legal issues challenge molecular forensic research, including personal and public concerns.^25^ Fortunately, in most large forensic pathology centres, which are often linked to tertiary academic institutions, forensic pathologists have established extensive FFPE tissue archives through routine histology casework. These archives are excellent and sometimes the only archives for conducting large retrospective genetic epidemiological studies.^9,15,20^

Until now, these forensic pathology FFPE tissue archives have mainly been unexploited; thus, the quality of DNA obtainable from these samples is unknown.^9,15,24^ African countries, particularly South Africa, have some of the highest numbers of unnatural deaths per year, compared to other countries in the world.^26,27^ Unfortunately, this increases the burden on the already-scarce Africa-practising forensic pathologists. Consequently, the increased caseload delays case investigation, prolonging formalin fixation times by days, weeks, and even months.^26,27,28^ According to recommended guidelines for optimised molecular testing, tissue samples should be fixed in 10% buffered formalin for 14 h – 24 h.^9,14^ Hence, most forensic pathology departments doubt the value and efficacy of their decades worth of FFPE tissue archives in the context of molecular diagnostic applications or research.^9,15,20^

This study aimed to determine the utility of FFPE tissue samples as a reliable source of genetic material for post-mortem molecular applications and diagnostics. Firstly, the study evaluated the influence of the procurement parameters, fixation time, and storage periods of the archived FFPE tissue samples on the quantity and quality of the DNA extracted and its efficiency for subsequent molecular applications. Secondly, the study aimed to determine which DNA extraction kits yield the best quality and quantity of FFPE DNA.

## Description of the intervention

### Ethical considerations

Ethics approval was obtained from the Faculty of Health Sciences Research Ethics Committee University of Pretoria (reference number 142/2014) to use the retrospective and prospective FFPE tissue samples and the control blood samples. The retention and use of tissues obtained at medico-legal post-mortem examinations were guided by the South African legislation (Inquests Act 58 of 1995, National Health Act 61 of 2003 and the Regulations Regarding the Rendering of Forensic Pathology Service R636). Thus, patient and family consent was not required. Healthy volunteers provided written informed consent before blood sample collection. All tissue and blood samples were assigned a case number, with no identifying features linked to any of these samples.

### Study location and design

This retrospective observational study was conducted in the Department of Forensic Medicine, the University of Pretoria, Pretoria, South Africa, from January 2015 to August 2016 using FFPE myocardial tissue samples obtained over 13 years from post-mortem examination of sudden unexplained infant deaths in the Pretoria Medico-Legal Laboratory. The samples were stored for further ancillary investigations to determine the cause of death, including genetic testing for genes linked to inherited cardiac arrhythmogenic disorders and sudden unexplained infant deaths, such as *SCN5A*.

### Specimen description

The study included a total of 58 FFPE myocardial tissue samples. These FFPE tissue samples were obtained from sudden unexplained infant death complete post-mortem investigations between January 2002 and January 2015 and identified from the Department of Forensic Medicine electronic repository. Additionally, in January 2015, two prospective myocardial tissue samples (samples 59 and 60) were also obtained from autopsy and, starting in March 2015, venous blood from nine healthy volunteers was drawn into two 5 mL ethylenediaminetetraacetic acid tubes.

### Specimen processing

When retained, the archived FFPE myocardial tissue samples were immediately fixed in 10% neutral buffered formalin solution per routine and processed using the Shandon Pathcentre from Thermo Scientific (Waltham, Massachusetts, United States) and the Tissue-TEK^®^ TEC from Sakura Finetek (Torrance, California, United States). The FFPE processing included dehydration in ethanol, clearing in xylene, and embedding in paraffin blocks.

When obtained, the prospective myocardial tissue samples (samples 59 and 60) were immediately fixed in formalin for 14 h – 24 h, per Qiagen protocol. Subsequently, they were cleared in xylene, embedded in paraffin blocks (following the same procedure as the archived samples), and stored for a month.

All FFPE tissue samples were cut into 20 μm thick sections using a microtome (Leica Biosystems, Wetzlar, Germany). The first three to four cuts of each FFPE specimen were discarded because the sample surface had been exposed to air. Then, to ensure sufficiency, six to eight sections of each sample were put into a well-labelled sterile 1.5 mL microcentrifuge tube for DNA extraction. During this specimen preparation process, the microtome’s blade and the cutting surface were cleaned with 100% ethanol between each specimen to prevent cross-contamination.

### Deoxyribonucleic acid extraction

Deoxyribonucleic acid was extracted from all FFPE samples (archived and prospective) using two different extraction kits; the QIAamp DNA FFPE tissue kit (Qiagen, Hilden, Germany) and the Isolate П FFPE DNA kit (Bioline, London, United Kingdom). Thus, two DNA extraction procedures were performed on every FFPE tissue sample. The two kits differed in the type of deparaffinisation solution and incubation time at 90 °C for the reversal of DNA cross-linking. The QIAamp DNA FFPE tissue kit used standard Qiagen-provided deparaffinisation solution and required an incubation time of 2 h at 90 °C, while the Isolate П FFPE DNA kit used xylene and incubation of 1 h at 90 °C.^29^

For the blood samples drawn into two 5 mL ethylenediaminetetraacetic acid tubes, the buffy coat was isolated (200 μL) and stored at – 80 °C until DNA extraction using the QIAamp DNA Blood Mini Kit (Qiagen, Hilden, Germany) per manufacturer’s instructions.

Further on, the concentration and purity of all DNA samples (archived FFPE, prospective FFPE and blood) were determined spectrophotometrically (NanoDrop, Thermo Scientific [Waltham, Massachusetts, United States]). For this study, a DNA concentration range of 40 ng/μL – 75 ng/μL and a purity ratio above 1.75 were deemed sufficient.

### High-resolution melt real-time PCR and sequencing

The DNAs were diluted to concentrations between 40 ng/μL and 75 ng/μL. Thirty-four amplicon primer pairs (online Supplementary Document Table 1) for the *SCN5A* gene^30^ generating 152 base pairs (bp) to 514 bp amplicon sizes amplified all extracted DNA (60 FFPE tissue samples: two prospective and 58 archived; and nine blood samples). Amplification was by high-resolution melt real-time PCR using SensiFast high resolution melt master mix (Bioline, London, United Kingdom) on the RotorGene Q (Qiagen, Hilden, Germany). Successful real-time PCR results had sigmoidal amplification and single melt curves. Afterwards, the PCR amplicons were Sanger sequenced by Inqaba Biotec, Pretoria, South Africa. Sequencing chromatograms were analysed using CLC Main Workbench 5 software (CLC Bio^®^, Aarhus, Denmark). A successful Sanger sequencing had evenly spaced peaks and a minimal noise chromatogram.

### Analysis

The tissue retention date and the 10% formalin fixation duration for each FFPE tissue sample (online Supplementary Document Table 2) were used for Student’s t-test in Microsoft Excel 2013 (Redmond, Washington, United States) to determine a possible correlation with the quality of PCR amplifiability.

## Lessons learnt

### Deoxyribonucleic acid yield

Deoxyribonucleic acid extracted from all nine control blood samples yielded concentrations ranging from 15 ng/μL to 56 ng/μL, with sufficient 260/280 ratios between 1.75 and 2.0.

### Comparison of DNA yield obtained from two different extraction kits

The maximum period of tissue sample storage (embedded in paraffin blocks) was 13 years, while the minimum was one month. The minimum period of tissue fixation in formalin wax was two days and the maximum, 271 days. The average value obtained for the fixation period of all 58 tissue samples was 26.1 days, with a median of 11.5 days. Our findings showed an extensive delay in the department’s processing of FFPE tissue samples, particularly long tissue fixation in formalin (online Supplementary Document Table 2). These findings are in keeping with similar FFPE archive conditions reported by other forensic pathology departments practising in resource- and fund-limited settings.^9,15,16,24^

The Qiagen FFPE kit yielded much higher DNA concentrations, 50 ng/μL – 900 ng/μL. After extraction, an elution volume of 50 μL of DNA of each sample was obtained, with purity ratios between 1.7 and 2.1. The Bioline FFPE kit yielded lower DNA concentrations, 33 ng/μL – 137 ng/μL, with 260/280 ratios between 2.0 and 2.1 and an elution volume of 50 μL. For this study, a DNA concentration range of 40 ng/μL – 75 ng/μL and a purity ratio above 1.75 were deemed sufficient.

Although the two different kits yielded DNA concentrations of quite different ranges, no difference in PCR amplification results was observed. Furthermore, both kits’ PCR amplification quality correlated; thus, successful or failed/unsuccessful amplification was recorded for each tissue sample. Therefore, we concluded that the two kits yielded the same quality of extracted DNA and PCR amplification and, by extension, the exact molecular analysis results.

### Does size matter?

Amplification of all 34 amplicons, high resolution melt analysis, gel electrophoresis, and sequencing were successful for all DNA extracted from blood.

**FIGURE 1:**
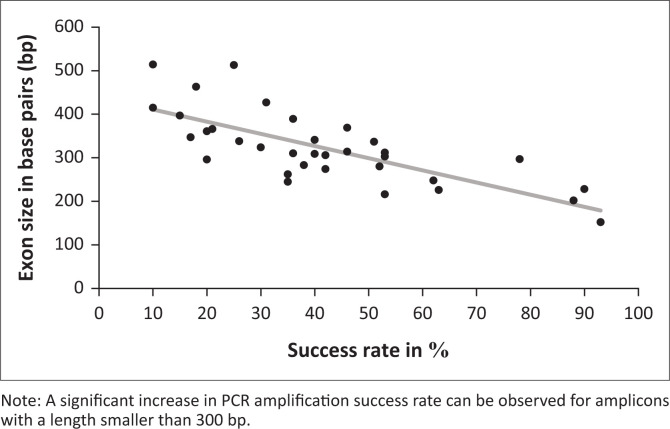
Effect of amplicon size on PCR amplification success rate of DNA extracted from FFPE tissue samples, Pretoria, South Africa, June 2015 – May 2016 (online Supplementary Document Table 3).

**FIGURE 2:**
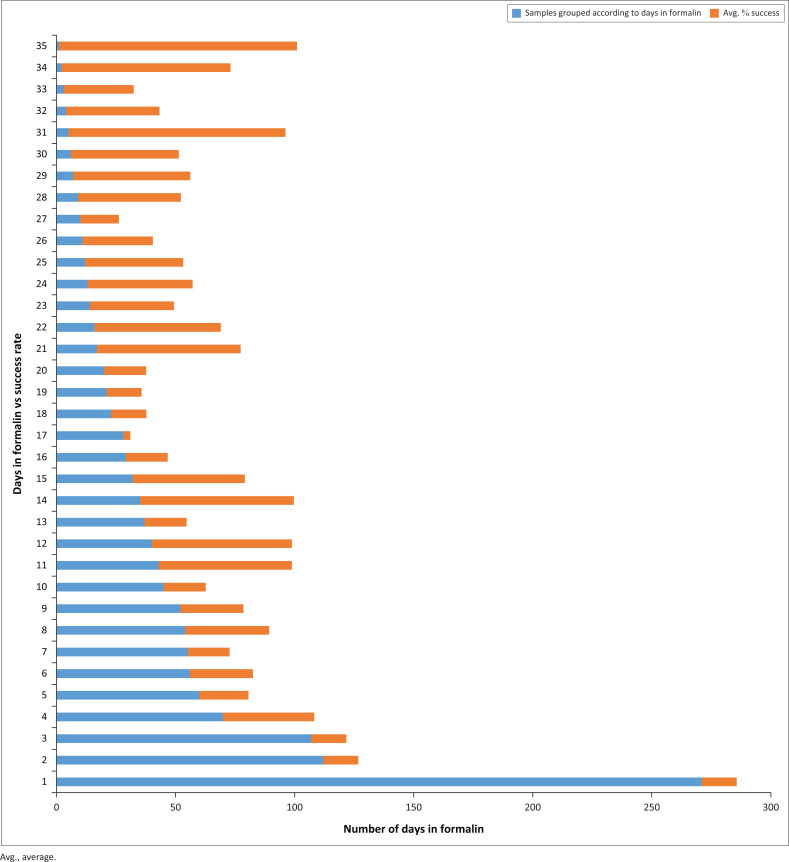
Laboratory results showing the association between a prolonged formalin fixation period and a decline in PCR amplification success rate, Pretoria, South Africa, March 2015 – July 2016 (online Supplementary Document Table 2).

Polymerase chain reaction amplified several amplicons from the FFPE DNA; however, there was a significant reduction in the amplification of amplicons with a length greater than 300 bp (Figure 1 and online Supplementary Document Table 3). In contrast to Vitosevic et al.,^31^ our study (as indicated in online Supplementary Document Table 2) did not find a correlation between longer sample storage time and PCR amplification failure, as successful amplification was observed for samples stored between 1 month and 13 years. Instead, we found that prolonged periods of formalin fixation, which causes DNA fragmentation, correlated with failed PCR amplification (Figure 2).

### Formalin fixation time

Approximately half of the FFPE DNA samples produced large amplicons; an association existed between successful amplification and a shorter (approximately four days) formalin fixation duration (Figure 2, online Supplementary Document Table 2 and online Supplementary Document Table 4). Converse to archived FFPE DNA, which had been fixed in formalin for a period exceeding 24 h, the two prospective FFPE samples (fixed in formalin for only 24 h) yielded all 34 targeted amplicons, including a 514 bp amplicon (the largest amplicon). Only four retrospective FFPE DNA samples yielded the 514 bp amplicon. On analysis, these four FFPE DNA samples, all stored for six years, were subjected to shorter periods of formalin fixation (Figure 1 and online Supplementary Document Table 2).

This bar graph shows the significant decrease in successful PCR amplification by using DNA extracted from FFPE tissue samples that have been fixated in formalin for periods longer than the prescribed 24 h. DNA samples extracted from those FFPE tissue samples which adhered to the prescribed 24-h formalin fixation illustrates a 100% success rate in PCR amplification. Samples were grouped according to days in formalin (see online Supplementary Document Table 4).

### Downstream applications

Sanger sequencing of amplicons was successful for both blood and FFPE tissue samples. Formalin-fixed paraffin-embedded tissue sequences that showed variations upon alignment to the reference sequence were re-amplified and re-sequenced to validate these variations and exclude the possibility of DNA cross-linking. Results showed no aberrant variations in FFPE DNA samples compared to amplicons resulting from blood DNA, indicating that the fixation method does not seem to increase the chance of interpreting these as legitimate variations.

## Recommendations

### Cut your losses?

This study showed that DNA extracted from FFPE tissue samples could successfully be both amplified and sequenced. However, it is essential to note that DNA measurements (concentration and purity ratio) obtained using a spectrophotometer do not assure successful PCR amplification and sequencing because it does not measure the extent of DNA fragmentation. Commercially available kits, such as the Quantifiler^®^ Trio DNA Quantification kit, are designed to detect and help overcome external factors affecting PCR amplifiable quality. However, in a resource-poor setting, a spectrophotometer, which most laboratories have, will indicate the ‘usability’ of extracted DNA.^32^ Polymerase chain reaction success was limited to amplicons smaller than 300 bp in cases of prolonged formalin fixation. Thus, FFPE samples could, and should, still be used in molecular diagnostics or research, as long as the primers are designed to generate PCR amplicons not exceeding 300 bp.

Our study confirmed that FFPE samples are still useful for molecular forensics and diagnostics despite inadequate sample preparation. Thus far, FFPE tissue archives in most African forensic pathology departments have been an underexploited resource for conducting large retrospective and prospective genetic epidemiological studies.^14,15,16^ It is time to utilise these resources at our disposal to the advantage of the African population. The most significant benefit of post-mortem molecular testing is the high disease-specific diagnostic, therapeutic, and prognostic benefits derivable from subsequent genetic analysis.^1,2,3^ Understanding the genetics of inherited diseases specific to African populations informs the development of more meaningful and relevant risk stratification techniques.^3,15,33^ This includes the development of more targeted molecular tests for various challenges in the forensic setting, including population genetics, human identification, and post-mortem interval estimation.^9,15^
